# Charging Effect by Fluorine-Treatment and Recess Gate for Enhancement-Mode on AlGaN/GaN High Electron Mobility Transistors

**DOI:** 10.3390/nano10112116

**Published:** 2020-10-24

**Authors:** Soo Cheol Kang, Hyun-Wook Jung, Sung-Jae Chang, Seung Mo Kim, Sang Kyung Lee, Byoung Hun Lee, Haecheon Kim, Youn-Sub Noh, Sang-Heung Lee, Seong-Il Kim, Ho-Kyun Ahn, Jong-Won Lim

**Affiliations:** 1 DMC Convergence Research Department, Electronics and Telecommunications Research Institute (ETRI), Daejeon 34129, Korea; hujung@etri.re.kr (H.-W.J.); sjchang@etri.re.kr (S.-J.C.); khc@etri.re.kr (H.K.); nohys@etri.re.kr (Y.-S.N.); shl@etri.re.kr (S.-H.L.); sikim@etri.re.kr (S.-I.K.); hkahn@etri.re.kr (H.-K.A.); jwlim@etri.re.kr (J.-W.L.); 2 Center for Emerging Electronic Devices and Systems (CEEDS), Gwangju Institute of Science and Technology (GIST), Gwangju 61005, Korea; kimsm2110@gist.ac.kr (S.M.K.); leesk@gist.ac.kr (S.K.L.); bhl@gist.ac.kr (B.H.L.)

**Keywords:** AlGaN/GaN HEMTs, enhancement-mode, fluorinated-gate, recessed gate

## Abstract

An enhancement-mode AlGaN/GaN metal-insulator-semiconductor high-electron- mobility-transistor was fabricated using a recess gate and CF_4_ plasma treatment to investigate its reliable applicability to high-power devices and circuits. The fluorinated-gate device showed hysteresis during the DC current-voltage measurement, and the polarity and magnitude of hysteresis depend on the drain voltage. The hysteresis phenomenon is due to the electron trapping at the Al_2_O_3_/AlGaN interface and charging times longer than milliseconds were obtained by pulse I-V measurement. In addition, the subthreshold slope of the fluorinated-gate device was increased after the positive gate bias stress because of the two-dimensional electron gas reduction by ionized fluorine. Our systematic observation revealed that the effect of fluorine ions should be considered for the design of AlGaN/GaN power circuits.

## 1. Introduction

AlGaN/GaN high electron mobility transistors (HEMTs) have recently demonstrated to be excellent devices for high-frequency and high-power electronics, thanks to the high breakdown voltage [1] and the low on-state resistance [2] and gate leakage [3]. The AlGaN/GaN HEMTs show normally on operation, since the two-dimensional electron gas (2DEG) channel is generated by the spontaneous and piezoelectric polarization at the AlGaN/GaN hetero-interface [4]. Various methods for enhancement mode (E-mode) device manufacturing through modulating the threshold voltage (V_T_) have been studied for the reduction in power consumption and circuit producing, such as an inverter using a depletion mode (D-mode) and an E-mode device [5,6,7,8,9]. The AlGaN barrier recess and fluorine ion implantation methods had been widely studied to shift the V_T_ in the positive direction.

Although these methods modulate the V_T_, the gate modifications introduce side effects resulting from the surface damage during the device fabrication process [10,11,12], and the electrical and frequency characteristics are degraded due to the poor interface conditions [13]. Various methods have been studied to improve the recessed interface condition [14,15]. However, hysteresis and V_T_ instability remain critical issues so far. In this perspective, it is necessary to better understand the degradation mechanisms and the trends of changing electrical properties related to charge trapping for reliable application.

In this study, the V_T_ was modulated by the gate recess and fluorine treatment employing CF_4_ plasma. The fluorinated-gate device (7-nm thick gate recess + fluorine plasma treatment) exhibited more positive-shifted V_T_ compared to the recess only gate device (7-nm thick gate recess). However, hysteresis of the fluorinated-gate device occurred during the DC current-voltage (I-V) measurement and the hysteresis depended on drain voltage (V_D_). We demonstrated that the cause of hysteresis was the charge trapping/detrapping effect introduced at the dielectric (Al_2_O_3_) and fluorinated AlGaN interface and gate leakage characteristics corresponding to V_D_. The increase in the subthreshold slope (SS) of the fluorinated-gate device after positive gate bias stress indicated the degradation in the AlGaN/GaN interface and the decrease in 2DEG density. As a result, we have revealed the effect of fluorine treatment on the dielectric/AlGaN and AlGaN/GaN interfaces through experimental evidence.

## 2. Device Fabrication and Electrical Characterization

The AlGaN/GaN heterostructure was grown by metal-organic chemical vapor deposition (MOCVD) on a sapphire substrate, which consists of a 21-nm AlGaN (26% Al) barrier with a 2-nm GaN cap layer. The Ti/Al/Ni/Au ohmic contact was formed by an E-beam evaporator and annealed at 900 °C for 150 s. After device isolation via phosphorus implantation, a 50-nm SiN passivation layer was deposited by plasma-enhanced chemical vapor deposition (PECVD).

**Figure 1:**
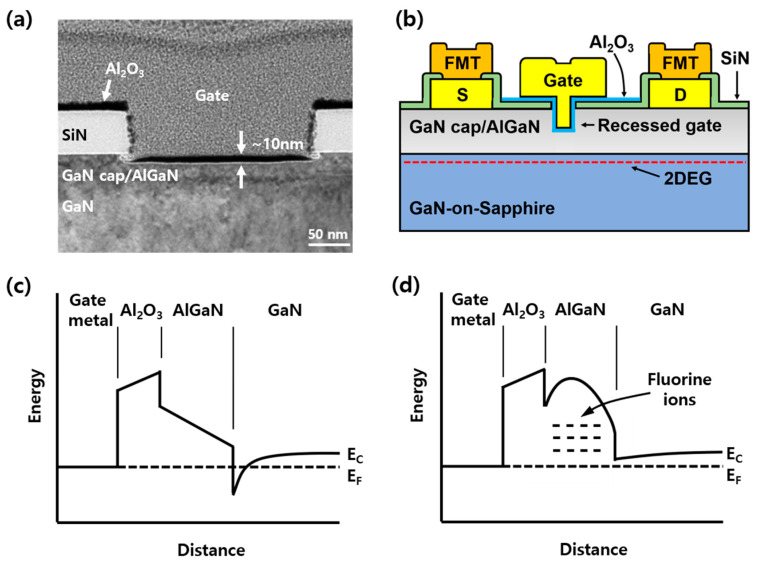
The fabricated AlGaN/GaN MIS-HEMTs on sapphire substrate. Recessed gate was formed under gate region for V_T_ modulation. (**a**) The TEM image of the recessed gate. The AlGaN layer was etched by the digital etch process in 15 cycles. (**b**) Schematic cross-section of the processed device. (**c**) Band diagram of MIS-HEMTs under equilibrium condition. (**d**) Band diagram of MIS-HEMTs after CF_4_ treatment under equilibrium condition.

Subsequently, the gate recess area was defined by electron-beam (E-beam) lithography and then the SiN was removed via an inductively-coupled plasma (ICP) etch. In order to obtain the etch rate of the gate region using a digital etch process, O_2_ plasma ashing (3 min) and 1:10 hydrochloric acid (HCl) dipping (1 min) were repeated 15 times in the test sample. An etch rate of 6.6 Å/cycle was obtained (in Figure 1a). The digital etches of 10 and 21 cycles were conducted with the same etch condition and each device had an etch depth of about 7 and 14 nm. For the fabrication of fluorinated-gate devices, CF_4_ plasma using reactive ion etching (RIE) was carried out for 30 s on the 7-nm recessed device. After that, Al_2_O_3_ with the thickness of 10 nm was deposited at 300 °C by atomic layer deposition (ALD). A T-shaped Ni/Au gate electrode was deposited by an E-beam evaporator. D-mode metal-insulator-semiconductor HEMTs (MIS-HEMTs) without gate recess and fluorine treatment were also prepared as a reference device. Figure 1b illustrates the cross-sectional view of the fabricated E-mode Al_2_O_3_/AlGaN/GaN MIS-HEMTs with a recessed gate. The fabricated device geometries are L_G_ = 0.25 μm, W_G_ = 50 μm, L_GD_ = 3.5 μm and L_GS_ = 1 μm. The DC I–V, pulse I–V measurement and stress application (for repetition of stress and measurement) were performed using a Keithley 4200-SCS parameter analyzer. The delay between the stress and measurement was less than tens of milliseconds. Capacitance of the device was measured using an Agilent 4294A impedance analyzer. The voltage was applied to the gate while the source and drain were grounded.

## 3. Results and Discussion

**Figure 2:**
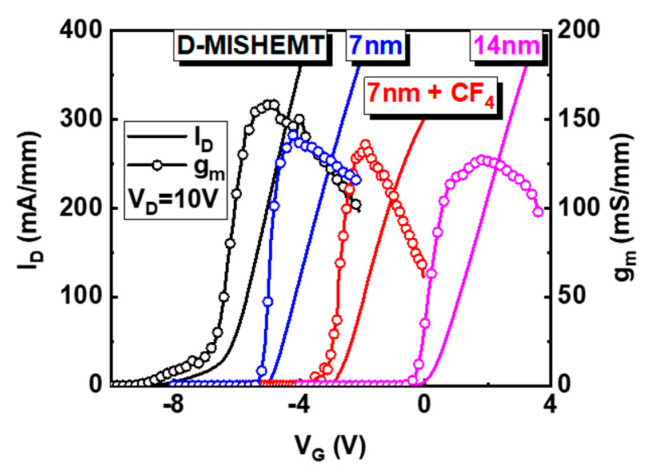
The I-V transfer curves (solid line) and transconductance (open circle line) of AlGaN/GaN MIS-HEMTs with various gate conditions at V_D_ = 10 V. Black, blue, red and pink colors indicate the D-mode device, 7-nm gate recessed, 7-nm gate recessed and CF_4_ treatment and 14-nm gate recessed device, respectively.

Figure 2 shows the I-V transfer curves and transconductance (g_m_) of AlGaN/GaN MIS-HEMTs with various gate condition. The V_T_ moves in the positive direction from the V_T_ of D-MIS-HEMT after the 7-nm gate recess and fluorine treatment, and the V_T_ further shifted after the 14-nm gate recess. The V_T_ depends on the barrier thickness, since the 2DEG concentration and electron mobility reduce with the decrease in AlGaN barrier thickness [16]. The fluorine treatment modulates the V_T_ by incorporating negatively charged fluorine ions into the AlGaN barrier. As a result, the V_T_ of D-MIS-HEMT, 7-nm recess, 7-nm recess + CF_4_ and 14-nm recess were −6.5, −5.2, −2.5 and +0.5 V, respectively. The V_T_ can be expressed as an Equation (1) [17]. The t_b_ denotes the barrier thickness, φ_b_ is the barrier height, *q* is the electron charge, σ_p_ is the 2DEG sheet charge density, N_F_ is the density of ionized fluorine atoms, X_F_ is the triangular fluorine ion distribution peaked at the surface and extending to a given depth and ε and ΔE_c_ are the dielectric constant and conduction band offset, respectively.

(1) $$V_{T}=\;\varphi_{b}\;-\;\left[\frac{q\left(\sigma_{p}t_{b}-\frac{N_{F}X_{F}^{2}}{6}\right)}{\varepsilon }\right]\;-\;\Delta E_{c}$$

**Figure 3:**
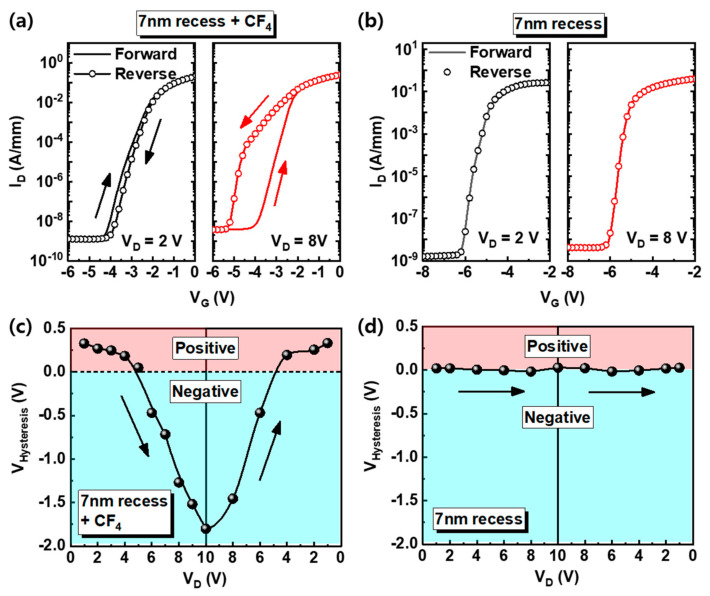
(**a**,**b**) The I-V hysteresis curves of 7-nm recess + CF_4_ and 7-nm recess devices measured at V_D_ = 2 V and 8 V, respectively. The solid line and circle line indicate the forward and reverse sweep, respectively. (**c**,**d**) The voltage difference of 7-nm recess + CF_4_ and 7-nm recess devices by hysteresis at various V_D_. Red and blue regions show the positive and negative direction of hysteresis, respectively.

However, the g_m_ of the 7-nm recess + CF_4_ device decreased sharply after maximum value in comparison with other devices. It is attributed to the electron trap in the AlGaN barrier under the gate region by the fluorine treatment [18]. The 7-nm recess + CF_4_ device shows hysteresis and it depends on the V_D_, as shown in Figure 3a. The characteristics of recessed gate (7-nm recess) and fluorinated-gate devices (7 nm-recess + CF_4_) were discussed to compare the fluorine treatment effect. For the hysteresis and V_D_ effect observation, the V_G_ increased in the forward direction (from the off-state to the on-state) and then decreased again in the reverse direction (from the on-state to the off-state) during the DC I-V measurement for various V_D_. When the V_D_ is increased from 1 V to 10 V and returned to 1 V, the hysteresis magnitudes (V_Hysteresis_) are almost symmetric. The V_Hysteresis_ is the V_G_ difference at a specific I_D_ (= 1 × 10^−7^ A/mm). It shows that the DC I-V measurement does not cause permanent charging. However, when the V_D_ is higher than 5 V, the direction of hysteresis becomes negative and the V_Hysteresis_ enlarges with increasing V_D_, as shown in Figure 3c. On the contrary, when the V_D_ is lower than 5 V, the direction of hysteresis is positive and the V_Hysteresis_ is relatively small. The negligible hysteresis of recessed gate device without fluorine treatment was achieved (Figure 3b,d).

**Figure 4:**
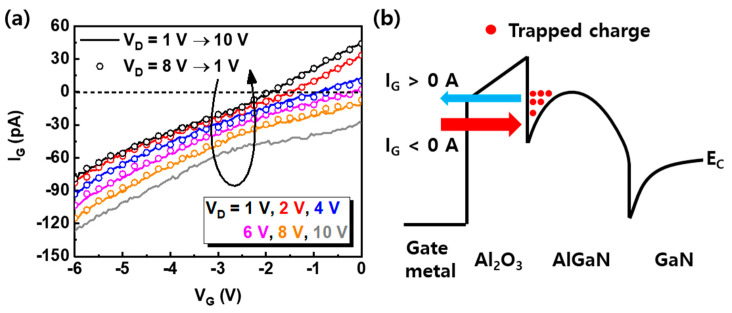
(**a**) The gate leakage current of the 7-nm recess + CF_4_ device for various V_D_. The solid lines and open circles reflect the increase in V_D_ and decrease of V_D_, respectively. (**b**) The band diagram of the 7-nm recess + CF_4_ device. The blue and red arrows indicate the flow of gate current. The trapped charges at the dielectric/AlGaN interface reduce by positive gate current (V_D_ < 5 V) and the trapped charges increase by negative gate current (V_D_ > 5 V).

The V_D_ dependence of hysteresis in the fluorinated-gate device was investigated in more detail by comparing the gate leakage current (I_G_) as a function of V_D_ in Figure 4a, and the band diagram and I_G_ flow direction are represented in Figure 4b. The I_G_ also depends on the V_D_, and when the V_D_ is larger than 6 V, the polarity of the I_G_ is negative. This means that the electrons are trapped in the Al_2_O_3_/fluorinated-AlGaN barrier as they move from the gate metal to the channel along the I_G_, and the V_T_ is negatively shifted. However, when the V_D_ is in between −2 V and 6 V, the polarity of I_G_ is positive, showing that some of electrons are detrapped from the Al_2_O_3_/fluorinated-AlGaN barrier to the gate metal and the V_T_ is slightly shifted to the positive direction.

**Figure 5:**
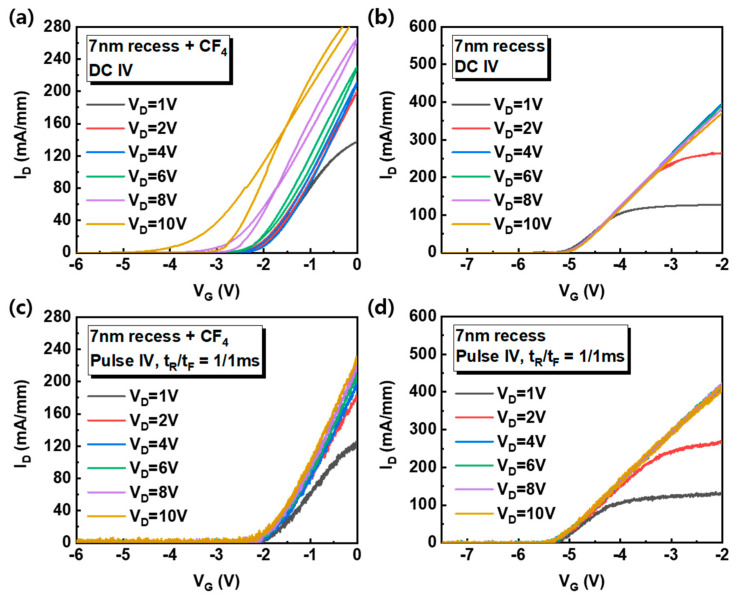
(**a**,**b**) The dual swept DC I-V transfer curve of 7-nm recess + CF_4_ and 7-nm recess devices measured at various V_D_. (**c**,**d**) Pulsed I-V curve of 7-nm recess + CF_4_ and 7-nm recess devices measured for various V_D_. The rising and falling time of 1 ms was used for pulse measurement.

The charging time in the Al_2_O_3_/fluorinated-AlGaN barrier was studied through a triangular pulse I-V measurement with rising (t_R_) and falling time (t_F_) of 1 millisecond (ms). As shown in Figure 5a, the hysteresis and the V_D_ dependence of V_T_ were obtained only in the fluorinated-gate device, whereas insignificant hysteresis was achieved for pulse I-V measurements, and the V_T_ is hardly changed with V_D_ (Figure 5c). However, the transfer curve of recessed gate devices is almost identical regardless of the pulse I-V measurement (Figure 5b,d). Therefore, it implies that the electron trapping/detrapping in the Al_2_O_3_/fluorinated-AlGaN barrier is a very slow charging mechanism and the trapping/detrapping time is longer than ms.

**Figure 6:**
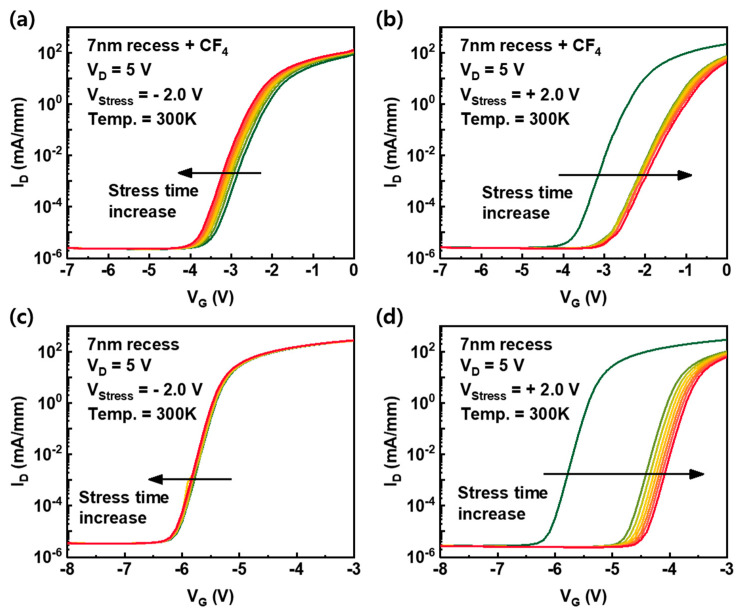
(**a**,**b**) The change in I-V transfer curves of the 7-nm recess + CF_4_ device under negative and positive gate bias stress conditions according to stress time, respectively. (**c**,**d**) The change in I-V transfer curves of the 7-nm recess device under negative and positive gate bias stress conditions, respectively.

**Figure 7:**
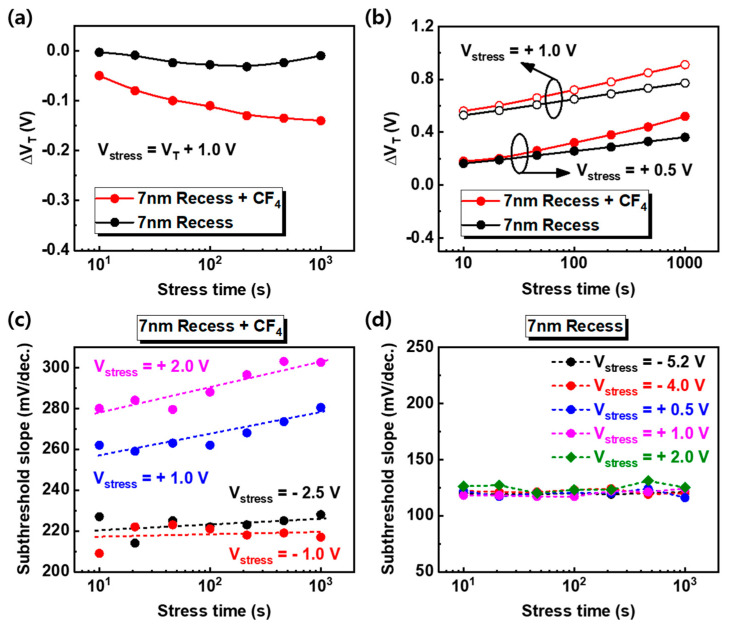
The V_T_ shift by gate bias stress according to stress time. A (**a**) negative (V_T_ + 1.0 V) and (**b**) positive gate bias stress were applied to the devices. The red and black circle line indicate the 7-nm recess + CF_4_ and 7-nm recess devices, respectively. (**c**,**d**) The change in the subthreshold slope for various gate bias stress corresponding to the stress time. The negative biases near the V_T_ and positive bias at 1.0 V and 2.0 V were used to estimate the degradation phenomenon.

To investigate the effect of fluorine in AlGaN under the gate bias stress, negative (−2 V) and positive (+2 V) gate bias stresses were applied to devices. Although the V_T_ of devices is different, the same stress voltage was introduced to compare the effect of the stress polarity (Figure 6). The V_D_ of 5 V was used as a condition to monitor the electrical characteristics of the devices and to minimize the charging effects that occurred during DC I-V measurements. The direction of V_T_ shift under the negative gate bias stress is negative; by contrast, the positive direction of V_T_ shift under the positive gate bias stress is positive, and this phenomenon is the same for both devices. It indicates the trapping (or detrapping) of electrons under the negative (or positive) gate bias stress at the AlGaN/GaN barrier. The different directions of V_T_ shift under negative and positive gate bias stress have been analyzed with the same mechanism in many other studies [19,20,21]. However, in the fluorinated-gate device, the V_T_ moved more, even under other stress conditions (V_stress_ = V_T_ + 1.0 V and V_stress_ = +0.5, +1.0 V), as shown in Figure 7a,b, due to the plasma-induced surface damage of the AlGaN barrier during fluorine treatment. In addition, the SS of the fluorinated-gate device is higher than that of the recessed gate device before stress. This indicates a higher interface state and is further evidence of the AlGaN surface damage caused by the CF_4_ plasma process. Furthermore, the SS of the fluorinated-gate device increased with stress time under the positive gate bias stress, whereas the SS of the recessed gate device without fluorine hardly changed with stress time, even under negative and positive gate bias stress. To observe in more detail, the change in SS is represented in Figure 7c,d. There are two possible origins of the SS increase after gate bias stress. One is the 2DEG reduction. The accelerated electrons from the channel under the positive gate bias stress induce the ionization of fluorine in the AlGaN barrier [22], and the ionized fluorine atoms are capable of forming bonds with gallium atoms with dangling bonds at the interface. The formation of Ga–F bonds is able to weaken the 2DEG channel and AlGaN/GaN interface [23]. Another is the degradation of the AlGaN/GaN interfacial layer by accelerated electrons under positive gate bias stress. For metal-oxide-semiconductor field-effect transistors (MOSFETs), the increase in SS primarily reflects the degradation of the interfacial layer [24,25]. However, the recessed gate device without fluorine did not show an SS change, even under the positive gate bias stress. Therefore, the Ga–F bonding mechanism makes more sense than the interface degradation.

**Figure 8:**
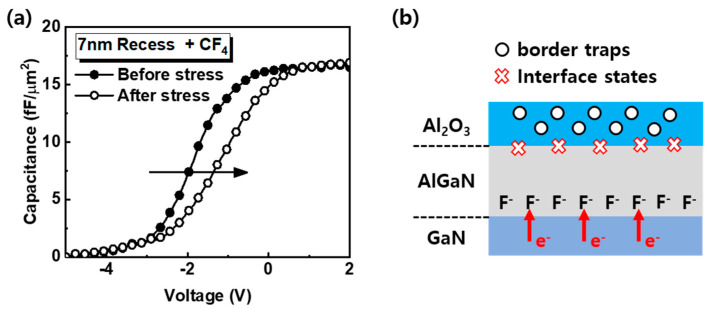
(**a**) The change in capacitance of the 7-nm recess + CF_4_ device before and after positive gate bias stress for 1000 s. The closed circle and opened circle indicate the capacitance before and after stress, respectively. (**b**) Schematic representation of the model used to explain the V_T_ shift of the 7-nm recess + CF_4_ device. The black circle and red X represent the border traps and interface states, respectively.

The capacitance–voltage (C–V) curve was measured before and after positive gate bias stress at 1 MHz, as shown in Figure 8a, and the C–V curve moved to a positive direction and the slope of the C–V curve lowered. AlGaN/GaN interface degradation and reduction in 2DEG after positive gate bias stress is also shown. We represent the schematic degradation model of fluorinated-gate device in Figure 8b. At the Al_2_O_3_/AlGaN interface, the trapped charges in the border trap site and/or interface state induce hysteresis during DC I-V measurement. The fluorine ions ionized by accelerated electrons by positive gate bias, and formation of the Ga–F bonds induces the reduction in 2DEG at the AlGaN/GaN barrier.

## 4. Conclusions

An E-mode AlGaN/GaN HEMT was fabricated employing a gate recess and CF_4_ plasma treatment, and the effect of fluorine in the device was investigated using an analysis of electrical characteristics. The fluorinated-gate device showed hysteresis during DC I-V measurement, which depends on the V_D_. The pulse I-V measurement was used to observe the charge trapping/detrapping effect of fluorine, and the charging time is longer than a few ms. It was also figured out that the SS of the fluorinated-gate device increased due to the 2DEG reduction by fluorine ions and the formation of Ga–F bonds after positive gate bias stress. We highlight that the impact of fluorine ions should be taken into account for circuit design when the E-mode device is employed.

## References

[1] Dora Y., Chakraborty A., Mccarthy L., Keller S., Denbaars S.P., Mishra U.K. (2006). High Breakdown Voltage Achieved on AlGaN/GaN HEMTs With Integrated Slant Field Plates. IEEE Electron Device Lett..

[2] Moens P., Banerjee A., Uren M.J., Meneghini M., Karboyan S., Chatterjee I., Vanmeerbeek P., Cäsar M., Liu C., Salih A. Impact of buffer leakage on intrinsic reliability of 650V AlGaN/GaN HEMTs. Proceedings of the 2015 IEEE International Electron Devices Meeting (IEDM).

[3] Ando Y., Kaneki S., Hashizume T. (2019). Improved operation stability of Al_2_O_3_/AlGaN/GaN MOS high-electron-mobility transistors grown on GaN substrates. Appl. Phys. Express.

[4] Sippel J.C., Islam S.S., Mukheijee S.S. A physics-based analytical model of a GaN/AlGaN HEMT incorporating spontaneous and piezoelectric polarization. Proceedings of the Canadian Conference on Electrical and Computer Engineering 2004 (IEEE Cat. No.04CH37513).

[5] Chiu H.-C., Yang C.-W., Chen C.-H., Fu J.S., Chien F.-T. (2011). Characterization of enhancement-mode AlGaN/GaN high electron mobility transistor using N_2_O plasma oxidation technology. Appl. Phys. Lett..

[6] Palacios T., Suh C.-S., Chakraborty A., Keller S., DenBaars S.P., Mishra U.K. (2006). High-performance E-mode AlGaN/GaN HEMTs. IEEE Electron Device Lett..

[7] Majumdar S., Sahu C., Biswas D. Fabrication of E-mode InGaN/AlGaN/GaN HEMT using FIB based lithography. Proceedings of the 2017 IEEE Electron Devices Technology and Manufacturing Conference (EDTM).

[8] Lee F., Su L.-Y., Wang C.-H., Wu Y.-R., Huang J. (2015). Impact of Gate Metal on the Performance of p-GaN/AlGaN/GaN High Electron Mobility Transistors. IEEE Electron Device Lett..

[9] Dutta Gupta S., Soni A., Joshi V., Kumar J., Sengupta R., Khand H., Shankar B., Mohan N., Raghavan S., Bhat N. (2019). Positive Threshold Voltage Shift in AlGaN/GaN HEMTs and E-Mode Operation By Al*_x_*Ti_1−*x*_O Based Gate Stack Engineering. IEEE Trans. Electron Devices.

[10] Wu T.-L., Franco J., Marcon D., De Jaeger B., Bakeroot B., Stoffels S., Van Hove M., Groeseneken G., Decoutere S. (2016). Toward Understanding Positive Bias Temperature Instability in Fully Recessed-Gate GaN MISFETs. IEEE Trans. Electron Devices.

[11] Lagger P., Ostermaier C., Pobegen G., Pogany D. Towards understanding the origin of threshold voltage instability of AlGaN/GaN MIS-HEMTs. Proceedings of the 2012 International Electron Devices Meeting.

[12] Kim K.-W., Jung S.-D., Kim D.-S., Kang H.-S., Im K.-S., Oh J.-J., Ha J.-B., Shin J.-K., Lee J.-H. (2011). Effects of TMAH Treatment on Device Performance of Normally Off Al_2_O_3_/GaN MOSFET. IEEE Electron Device Lett..

[13] Jung H.-W., Chang S.-J., Do J.-W., Ahn H.-K., Cho K.-J., Kim J.-J., Kim S.-I., Min B.-G., Kim H., Yoon H.S. (2018). DC and RF Characteristics of Enhancement-Mode Al2O3/AlGaN/GaN MIS-HEMTs Fabricated by Shallow Recess Combined with Fluorine-Treatment and Deep Recess. ECS J. Solid State Sci. Technol..

[14] Wang Y., Wang M., Xie B., Wen C.P., Wang J., Hao Y., Wu W., Chen K.J., Shen B. (2013). High-Performance Normally-Off Al_2_O_3_/GaN MOSFET Using a Wet Etching-Based Gate Recess Technique. IEEE Electron Device Lett..

[15] Li S., Hu Q., Wang X., Li T., Li X., Wu Y. (2019). Improved Interface Properties and Dielectric Breakdown in Recessed AlGaN/GaN MOS-HEMTs Using HfSiO_x_ as Gate Dielectric. IEEE Electron Device Lett..

[16] Zhao Y., Wang C., Zheng X., Ma X., He Y., Liu K., Li A., Peng Y., Zhang C., Hao Y. (2020). Effects of recess depths on performance of AlGaN/GaN power MIS-HEMTs on the Si substrates and threshold voltage model of different recess depths for the using HfO_2_ gate insulator. Solid State Electron..

[17] Klein B.A., Douglas E.A., Armstrong A.M., Allerman A.A., Abate V.M., Fortune T.R., Baca A.G. (2019). Enhancement-mode Al0.85Ga0.15N/Al0.7Ga0.3N high electron mobility transistor with fluorine treatment. Appl. Phys. Lett..

[18] Du J., Chen N., Jiang Z., Bai Z., Liu Y., Liu Y., Yu Q. (2016). Study on transconductance non-linearity of AlGaN/GaN HEMTs considering acceptor-like traps in barrier layer under the gate. Solid State Electron..

[19] Meneghini M., Rossetto I., Bisi D., Ruzzarin M., Van Hove M., Stoffels S., Wu T.-L., Marcon D., Decoutere S., Meneghesso G. (2016). Negative Bias-Induced Threshold Voltage Instability in GaN-on-Si Power HEMTs. IEEE Electron Device Lett..

[20] Guo A., del Alamo J.A. (2017). Unified Mechanism for Positive- and Negative-Bias Temperature Instability in GaN MOSFETs. IEEE Trans. Electron Devices.

[21] Cheng L., Xu W., Pan D., Liang H., Wang R., Zhu Y., Ren F., Zhou D., Ye J., Chen D. (2020). Gate-first AlGaN/GaN HEMT technology for enhanced threshold voltage stability based on MOCVD-grown in situ SiNx. J. Phys. Appl. Phys..

[22] Ma C., Chen H., Zhou C., Huang S., Yuan L., Roberts J., Chen K.J. (2011). ON-state critical gate overdrive voltage for fluorine-implanted enhancement-mode AlGaN/GaN high electron mobility transistors. J. Appl. Phys..

[23] Huang S., Chen H., Chen K.J. (2010). Effects of the fluorine plasma treatment on the surface potential and Schottky barrier height of AlxGa1−xN/GaN heterostructures. Appl. Phys. Lett..

[24] Kang S.C., Kim S.M., Jung U., Kim Y., Park W., Lee B.H. (2019). Interface state degradation during AC positive bias temperature instability stress. Solid State Electron.

[25] Cho M., Roussel P., Kaczer B., Degraeve R., Franco J., Aoulaiche M., Chiarella T., Kauerauf T., Horiguchi N., Groeseneken G. (2013). Channel Hot Carrier Degradation Mechanism in Long/Short Channel $n$-FinFETs. IEEE Trans. Electron Devices.

